# Effectiveness of a mobile app-based educational intervention to treat internet gaming disorder among Iranian adolescents: study protocol for a randomized controlled trial

**DOI:** 10.1186/s13063-022-06131-0

**Published:** 2022-03-21

**Authors:** Amir H. Pakpour, Sara Fazeli, Isa Mohammadi Zeidi, Zainab Alimoradi, Mattias Georgsson, Anders Brostrom, Marc N. Potenza

**Affiliations:** 1 Social Determinants of Health Research Center, Research Institute for Prevention of Non-Communicable Diseases, Qazvin University of Medical Sciences, Qazvin, Iran; 2 Department of Nursing, School of Health and Welfare, Jönköping University, Jönköping, Sweden; 3 Student Research Committee, Qazvin University of Medical Sciences, Qazvin, Iran; 4 Department of Clinical Neurophysiology, Linköping University Hospital, Linköping, Sweden; 5 Departments of Psychiatry and Neuroscience and the Child Study Center, School of Medicine, Yale University, New Haven, CT USA; 6 Connecticut Council on Problem Gambling, Wethersfield, CT USA; 7 Connecticut Mental Health Center, New Haven, CT USA

**Keywords:** Internet gaming disorder, Transtheoretical model, Adolescents, Application, Randomized controlled trial, Cognitive-behavioral therapy, Addictive behaviors, Video games, Internet addiction disorder

## Abstract

**Background:**

The use of video games, a hobby for many teenagers in their leisure time, has brought with it a new potential for concerns. Internet gaming disorder (IGD) is a mental condition classified as a disorder due to addictive behaviors. It may include use of video games, both online and offline. Consequences of IGD may include introversion, social anxiety, mood swings, loneliness, sleep problems, behavioral problems, depression, low self-esteem, and increased violence. In order to design an app-based intervention for adolescents, a transtheoretical model (TTM) has been used. This widely used model in the field of behavioral change is also practical for health education programs. In addition, cognitive-behavioral therapy (CBT) has been used to make people more aware of their behaviors, feelings and thoughts and how to achieve behavioral change. The present study seeks to determine the effectiveness of this app-based intervention in in the treatment of IGD among adolescents.

**Method:**

In this single-blinded, randomized, controlled trial, 206 high-school adolescents aged 13 to 18 years in Qazvin city will be recruited. Eligible adolescents will be randomly assigned into intervention and control groups. Eight consecutive sessions delivered over 2 months and based on the TTM and CBT will be delivered through the `app (named HAPPYTEEN) to the intervention group. The control group will receive a sleep hygiene intervention (8 consecutive sessions for 2 months) via the app. Data collection tools include the Internet Gaming Disorder Scale, Insomnia Severity Index, Depression, Anxiety, and Stress Scales, Stages of Change Questionnaire, Decision Balance, and Self-Efficacy. The study measures will be completed at baseline, post intervention, and 1 month and 3 months after the intervention.

**Discussion:**

The results of this intervention could be used as adjunct therapy for adolescents with IGD.

**Trial registration:**

Clinical Trial Registration Center of Iran (IRCT) IRCT20181226042140N1. Registered on June 9, 2020.

**Supplementary Information:**

The online version contains supplementary material available at 10.1186/s13063-022-06131-0.

## Background

In the setting of dramatic growth of communication technologies, computer games as a social phenomenon often attract children and adolescents [1]. Some youth may experience difficulties regulating time spent playing video games, leading to addictive engagement [2, 3]. The 11th revision of the International Classification of Diseases (ICD-11) was officially adopted at the World Health Assembly [4]. Gaming disorder (GD) was included in the ICD-11 (and internet GD (IGD) was included in the 5th Edition of the Diagnostic and Statistical Manual (DSM-5®)) to define criteria for disordered patterns of gaming [4]. GD may include use of a variety of video games, whether online or offline, whereas IGD specifies the focus on gaming conducted online, often competitively in groups [5, 6]. Types and patterns of gaming may lie along a continuum from enjoyable to harmful and addictive [7].

Gaming in GD, like substance use in substance use disorders, may be motivated by desires to escape problems in life [8]. According to the American Psychiatric Association, IGD is characterized by a persistent pattern of behavior and frequent use of the internet to engage in online gaming, leading to significant disruption or discomfort over a 12-month period. Five out of 9 inclusion criteria are needed for diagnosis. These criteria include preoccupation, withdrawal, tolerance, unsuccessful attempts to limit time spent gaming, diminished interests in non-gaming activities, continued gaming despite adverse consequences, deceiving family members, therapists and others about durations of gaming, using online games to reduce negative moods, and jeopardizing relations or educational or occupational opportunities due to internet gaming [9].

In Iran, a study by the National Youth Organization shows that the number of people using the internet has grown by 70% of the population last year [10]. Zamani et al. found that of 564 seventh-graders, 17% experienced gaming problems [11], and another study reported that nearly 60% of 210 Iranian adolescents spend at least one or more hours a day playing video games [12]. More than 35% of people using the internet in Iran are youth/young adults, and the average time spent on the internet is generally 52 min per week [13].

Globally, individuals aged 16 to 21 years appear among the most vulnerable for developing gaming problems [14]. Approximately one in 21 adolescents may experience IGD [15]. However, prevalence estimates of IGD vary across jurisdictions [14]. In Spain, 0.6% of adolescents [16], and in the UK, 19.9% [17] experience gaming problems, and according to a study in Turkey, 4.5% of 16-year-olds met criteria for IGD [18]. Approximately 1.5–3% of adolescents aged 13–16 years in the Netherlands had IGD [19]. In East Asian countries, prevalence estimates may be higher [16], with males more frequently gaming and having gaming problems relative to females [4, 20]. This variability in prevalence estimates may reflect differences in assessment instruments, study populations, and the criteria for diagnosing IGD [7]. In Iran, the prevalence of gaming in children and adolescents is reported to range between 30% and 72% [21]. Another study found that 56% of young Iranians are very interested in computer games and use the internet in their spare time [21].

IGD may alter behavioral tendencies. For example, after prolonged gaming and IGD, people may experience introversion, social anxiety, mood swings, loneliness, sleep problems, behavioral problems, depression, aggression, and low self-esteem [22, 23]. However, longitudinal studies are needed to disentangle pre-existing vulnerabilities from consequences of gaming and IGD.

Successful educational interventions typically involve theoretical planning [24]. The use of behavior change models/theories may help in recognizing the factors affecting behavior change and facilitating successful intervention [25]. The transtheoretical model (TTM) is widely employed [26] and focuses on explaining how to change behavior over time [27]. This model has many applications for behavioral change, including for physical activity, smoking cessation, alcohol withdrawal and IGD among adolescents, youth, and adults [28, 29].

Few studies have used cognitive-behavioral therapy (CBT) in the treatment of IGD [30]. CBT has been used for many mental health-related illnesses including anxiety and depressive disorders and alcohol, drugs, gambling, and internet addictions [31–35]. CBT has demonstrated effectiveness in changing maladaptive behaviors [36, 37]. Due to the behavioral similarities between people who experience substance use and gambling disorders and those with IGD, some targeted domains of CBT for IGD may include stimulus control and learning of and enacting healthy coping responses when craving. Motivational understanding, self-monitoring, cognitive restructuring, and problem solving are also important considerations [30, 38, 39].

In recent years, psychological treatments have undergone considerable changes given the widespread availability of digital technologies including computers, the internet, mobile devices such as smartphones, and mobile software applications [40]. Many digital treatments are provided in the form of CBT [41]. Increased convenience, privacy, and trust in this method have increased demand for such treatments [42]. The internet is an emerging platform for mental health services worldwide [43–45]. The internet serves as a potential solution to geographic and transportation challenges and may facilitate the provision of mental health services to patients in their own homes. Internet-based CBT has shown promising results for addressing different mental problems [46–48]. Emerging evidence suggests that internet-based CBT is as effective as face-to-face CBT [48–52]. If the content of CBT-based technology that is disseminated via the internet, emails, or mobile phones is of good quality and has credible evidence-based efficacy and effectiveness, it may have widespread positive therapeutic effects and patient acceptance, similar to face-to-face CBT [53, 54]. Of note, given that gaming is often done online, appropriate precautions should be considered and used when conducting treatment for gaming problems via online platforms. Given the ubiquity of digital devices and their incorporation in many aspects of daily life for most people, such precautions will likely be beneficial in general for any intervention with IGD populations.

The study will aim to determine the effectiveness of an educational intervention based on the TTM model and CBT in the treatment of IGD in adolescents.

## Methods/design

### Objectives

The overall aim of this trial is to determine the effectiveness of an educational intervention based on the TTM model and CBT in the treatment of online gambling addiction through an app in adolescents.

More specifically the study aims are to compare in the active control and intervention groups over time (before and one and three months after):

1. Stages of change
2. Self-efficacy
3. The balance of decisional choices
4. The intentions to perform preventive behaviors relating to online gaming
5. Levels of stress, anxiety and depression
6. IGD severity
7. Gaming duration
8. Sleep health status

### Study design

The implementation of the educational program and intervention will be online by using an app called HAPPYTEEN.

**Fig 1:**
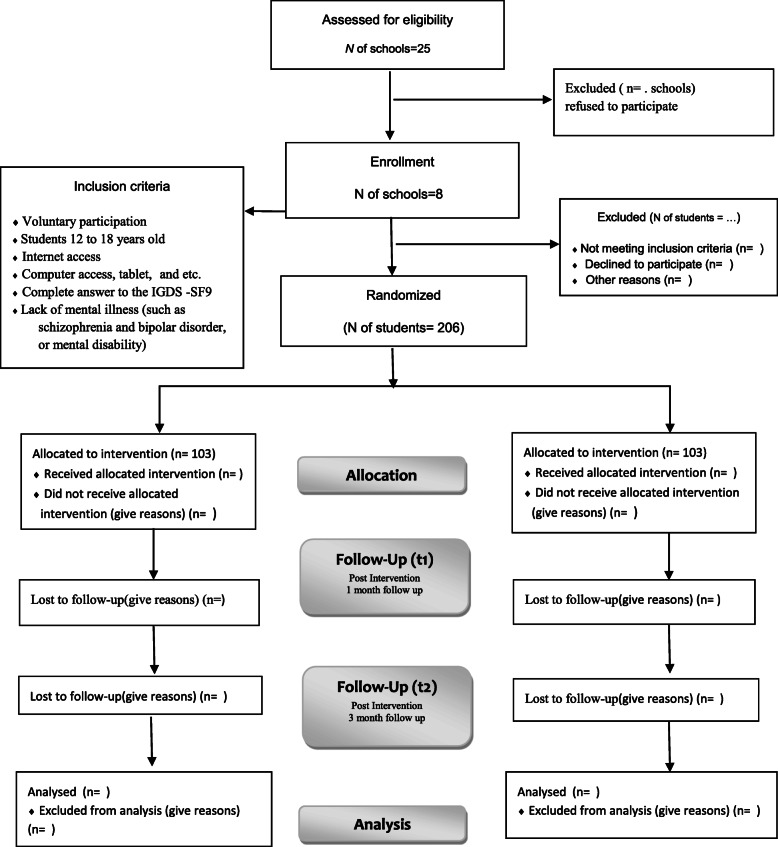
CONSORT flow diagram

The stages of this research will briefly include educational planning, intervention implementation, and evaluation at three times (before the intervention and at 1-month and 3-month follow-ups after the intervention). The study flow diagram is provided in Fig. 1.

In order to select the sample, a one-stage sampling method will be used. For this purpose, a list of secondary schools will be prepared. Then, out of 25 high schools, 8 will be randomly selected and screened for gaming disorder in all students who play computer games (both online and offline) using the nine-item Internet Gaming Disorder Scale - Short Form (IGDS-SF9). An online questionnaire will be provided to adolescent students through school information channels so that those who volunteer may participate in this research. Eligible adolescents (*n* = 206) will be randomly assigned into action control (*n* = 103) and intervention groups (*n* = 103). An online questionnaire will be provided to adolescent students through school information channels so that those who volunteer can participate in this research.

After approval of the ethics Committee in Research of Qazvin University of Medical Sciences (IR.QUMS.REC.1398.358), a letter will be sent to the General Department of Education of Qazvin Province to obtain all high school lists and permission to conduct the research. The first author will hold coordination meetings with school principals and explain the research clearly. After the schools’ agreement, an announcement of the study will be posted in the schools’ channels. During the COVID-19 pandemic, a specific application (the SHAD web) was developed by the ministry of education to be used for all schools for teaching, parental involvement, and other activities. Each school has this application. Screening questionnaires will be provided to the target group through these school information channels (i.e., the SHAD WEB). In addition, in all stages of the research, to ensure the participants' satisfaction, they will be provided with an explanation that all their answers and statements will be kept completely confidential and tracked by numbers. After reviewing eligibility criteria, eligible adolescents will complete baseline measures and then will be randomly divided into two groups (active control and intervention) as detailed above and below. The intervention group will receive an educational app (HAPPYTEEN) based on the TTM model and CBT. The intervention will be conducted weekly in eight sessions with an average duration of 30 min, while the active control group will receive an intervention on sleep hygiene. It should be noted that individual participants in both groups will be assigned a unique code as username (phone number or email) to enter the app. Initial feedback from an independent sample of adolescents found that the app was understandable and acceptable to adolescents. All participants’ activities (including opening each page, duration of reading each page, the number of logins) will be monitored and evaluated by an independent researcher to ensure adherence to treatment among participants. In the app user environment, participants can also communicate with the support team via email and phone message to share any questions, criticisms, and suggestions with an independent support team. Messages related to reminder training tips and reminders to answer weekly questions are possible through the app.

### Participants and procedure

#### Inclusion and exclusion criteria

Inclusion criteria for eligible adolescents to enter the trial will be as follows: voluntary participation of adolescents aged 13 to 18 years; studying in Abeyek high schools; full access to a computer or laptop, tablet or smartphone; internet access; scoring 32 or higher on the nine-item Internet Gaming Disorder Scale – Short Form (IGDS-SF9) [55]; and providing consent to participate in the study by both parents and adolescents. Adolescents with mental disabilities or severe mental health-related illness (such as schizophrenia and bipolar disorder) will be excluded. Moreover, the participants will be excluded if they do not receive more than 50% of the interventional materials. The procedure of recruitment and acquiring informed consent will be done by the second author (SFR).

#### Randomization

Recruitment will begin on June 1, 2021. After screening the adolescents and having eligible adolescents complete the questionnaires, randomization will be conducted. This study is a two-group, parallel, single-blind randomized controlled trial with a sample size of 206 adolescents. From a total of 25 schools, 8 schools will be randomly selected by simple randomization methodology. A list of schools will be provided by referring to the Department of Education. All high schools will be considered eligible to have the opportunity to reach students of all different socioeconomic levels. After checking the eligibility of the adolescents, of the 206 participants’ schools, 103 adolescents will be randomly assigned to the intervention group, and the other 103 adolescents randomly assigned to the active control group by simple randomization methodology. The simple randomization will be done to assign participants for study groups by an independent statistician with a 1:1 allocation.

From each school, the students selected will receive a special code through SPSS software, and after analyzing the data, they will be selected randomly through these codes.

Blindness will not be performed in study participants due to the different natures of the approaches in the active control and intervention groups, with one group receiving educational training on IGD and the other receiving educational information on sleep hygiene. Study administration and the statistician will be blinded to group allocation.

### Study interventions

#### Intervention condition

The TTM model consists of six domains including (1) stages of change, (2) process of changes, (3) decisional balance, (4) change level, (5) self-efficacy, and (6) temptation. In this study, we will focus on three domains, as described below. The first domain is stages of change. While changing behavior and achieving the desired behavior, it is important to consider different stages [27]. In the first stage (Stage 1: Precontemplation), due to lack of awareness or knowledge or experiences of failed attempts to change in the past, the person may not have enough motivation to consider behavioral change during the next 6 months. In the next stage (Stage 2: Contemplation), the person has the necessary knowledge regarding the results of performing the desired behavior and knows the benefits of performing the behavior and the costs of performing the behavior, but he/she may not have self-confidence or motivations to make changes in behavior during the next 6 months. In the third stage (Stage 3: Preparation), people may analyze more fully costs and benefits in performing the behavior. People in this stage value the benefits more than the obstacles, and by planning for the next 30 days, they typically feel one step closer to their goals. After the preparation stage, the person may be better able to resist temptations and persevere to adopt the new behavior. In the next stage (Stage 4: Action), a new behavior may be adopted by the person within the next 6 months. In the next stage (Stage 5: maintenance), positive changes in his/her lifestyle have typically occurred, and he/she has experienced a change in behavior for at least 6 months and a maximum of 5 years with more confidence and less temptation. The risk of unusual temptations and the consequences of false self-confidence may still precipitate relapse.

The second domain of the TTM model involves processes of changes. These processes include overt and covert activities that a person may use to progress through the stages of change. In other words, these include any activities that a person does to help modify the way he/she thinks, feels or behaves.

The next TTM domain considered is decisional balance. Decision-making is the process by which an individual considers the outcome of mental agreements (positive mental images, values, and beliefs) and oppositions to engage or not in behaviors. This domain is similar to that of perceived benefits and barriers in the health belief model [56]. The balance between perceived agreements and disagreements often fluctuates throughout stages of change.

Self-efficacy is the third domain considered for the intervention group. This domain, which comes from Bandura's theory of social learning, is a person’s self-efficacy and has a linear and positive relationship with the domain of stages of change. If a person goes to higher stage of change, his/her self-efficacy also increases [57].

The present study will focus on three domains of the TTM model (i.e., stages of change, self-efficacy and decision balance) in order to investigate the role of the TTM model in the treatment of IGD. One of the reasons for selecting these three domains is their success in statistically predicting individuals’ addictive gaming behaviors, which has been examined and demonstrated in a previous study [58].

Beside the TTM model, CBT will be used for our intervention. CBT is a form of psychotherapy that helps people understand thoughts and feelings that influence their behaviors. During treatment, a person learns how destructive or disturbing thoughts may have negative effects on their behaviors [59], and they may learn coping skills to manage their thoughts and feelings in a more healthy fashion [30].

After installing the app, each individual enters into the study with a use code that can be a mobile phone number or email that is recorded in the initial screening of each person in the study. Thereafter, a confirmation code is received via SMS.

The main page of the app consists of 4 sections: 1. content of training sessions, 2. exercises and weekly assignments, 3. questionnaires, and 4. a progress chart. The trainings are designed for 8 consecutive weeks (one session per week), and the approximate duration of each session is 30 min. Training includes written texts, videos, and audio files (relaxation music, relaxation exercise, and audio stories). Table 1 provides an overview of the content of each session in the intervention group (Screenshots of the app is provided in Additional file 1).

**Table 1:** Intervention content for the intervention group, based on cognitive behavioral therapy (CBT)

Session	Content
First	Introduction of the program, definition of IGD, types of video games, comparison of the prevalence of the disease in Iran and the world, consequences of online games, review of diagnostic criteria for online gaming disorder
Second	Learning to communicate with others, listening skills, bad communication characteristics, secrets of effective communication, teaching mindfulness, friendship and kindness, motivational video, homework presentation
Third	Identifying negative thoughts, replacing realistic thoughts, the impact of thoughts on emotions, learning coping skills with negative thoughts, video lectures, homework presentation
Fourth	Relationship between stress and IGD, definition of stress, types and symptoms of stress, identifying stressors, positive and negative strategies for coping with stress, video lectures related to the session and learning to breathe properly, audio story, homework presentation
Fifth	Relaxation training, mindfulness training, body scan and breathing along with audio files
Sixth	Review of past sessions, familiarity with the importance of problem solving, teaching problem solving techniques, motivational videos
Seventh	Review of past sessions, prevention of relapse, training to adopt a healthy lifestyle, providing solutions to prevent relapse of IGD, video lectures related to the session, homework presentation
Eighth	Summarize the content along with an overview of all past sessions

The training time for each person is counted through the app from the day the sessions start until the content of the next session is activated, which is 7 days later. A reminder message will be sent to each participant to announce each new session. The content of the sessions includes raising awareness and examining the impact of online gaming on people's physical and mental health. It also identifies and corrects misconceptions about online games. This training also includes learning self-control, coping responses, and how to overcome temptation.

The third part of the app includes questionnaires. Participants will answer questions at baseline, 1 month, and 3 months after the end of intervention.

The fourth part of the app aims to evaluate the effectiveness of the intervention in each session according to the weekly completion of the IGDS-SF9 incorporated into exercises and assignments. Participants can monitor their progress in the chart.

Behavior-change interventions are usually complex, complicating research efforts and implementation of practical applications. A method proposed by Michie et al. characterizes the reliability of interventions in terms of behavior change techniques (BCTs) [60]. BCTs are defined as visible, reproducible, and integral components of an intervention designed to change behavior. BCTs can be used alone or in combination and in different forms. We will use BCTs in intervention group to increase effectivity. Several BCTs have been used for this intervention in the app during each session. These BCTs are summarized in the Table 2.

**Table 2:** Defined behavior change techniques (BCTs) with links to objectives for the intervention group

BCT number	Name	Description
1.2	Problem solving/coping planning	In the content of the sixth session, it will be discussed that adolescents should make a list of problems that may occur during the day and even imagine the problems they are currently facing. They will be then instructed to choose the problem that is most important to them and define it clearly for themselves (for example: excessive use of online games). They will be then instructed to think about the consequences that playing online games has had on their health, or has changed their behavior with those around them, or has led them to stay up late at night. They will be then instructed to make a list of these and look for solutions to reduce time spent gaming, listing what comes to mind, how they can control their gaming time, and then finding solutions that they find desirable and easy to implement.
1.4	Action planning	In the sixth session, the importance of management of time spent playing online games will be discussed. Adolescents will receive instruction regarding how to manage their intention to keep their gaming time low, with a focus on making decisions about durations, locations and plans for reductions in gaming. The adolescents will be encouraged to decide how they will do this.
2.3	Self-monitoring of behavior	In the app progress chart section, from the first to the eighth week, adolescents will be asked to complete a 9-question Internet Game Disorder Questionnaire on weekly basis. The total score of the questionnaire will be presented in a graph. They will be able to see their progress on a weekly basis based on the answers provided and points they receive.
4.1	Instruction on how to perform a behavior	In all sessions, training regarding communication skills (the second week), identifying negative thoughts, effects of thoughts on emotions, learning alternative behaviors (the third week), stress management (the fourth week), and training in coping skills (the seventh week) will be used. For example, in the fifth week, relaxation techniques constitute one of the trainings used for participants to reduce stress and improve self-efficacy to overcome excessive use of online games.
5.1	Information about consequences	In the first week, a session will describe consequences of IGD on physical and mental health, resulting behavioral problems and financial costs that these games may create for adolescents.
5.4	Monitoring of emotional consequences	In the third week, participants will be informed about their situation by challenging their negative thoughts with a series of questions. Logical and useful alternative perspectives that they may adopt will be provided. Participants will be asked to write down events that they experience during the week according to the approach they have learned in the exercises section.
8.1	Behavioral practice/rehearsal	At the end of each session, adolescents will be asked to do their homework by referring to the exercises related to each session to stabilize their behavior to perform a new activity and observe a change in their behavior in the long term.
9.1	Credible source	Depending on the topic of each session, we will use the best and most expressive videos and music that emphasize the promotion of that behavior. In week 2, we will use the motivational video “Say Thank You.” In the fourth week, we will use a clip entitled, “The impact of stress on the body.”
9.2	Pros and cons	Outline the potential risks of problematic gaming (e.g.. neuroticism, aggression and hostility, sensation seeking, and attention deficit hyperactivity disorder). The adolescents will be asked to list the potential risks of problematic gaming and the potential benefits of non-problematic gaming (e.g., healthy brain stimulation)

The use of TTM model constructs in the intervention content of the HAPPYTEEN app is as follows:

##### Stages 1 and 2: Precontemplation and contemplation

The app intervention has been personalized based on the adolescent’s place in stages of change. For example, for those adolescents who are identified in precontemplation (Stage 1) and contemplation (Stage 2) stages, the app will direct them to start the intervention from session 1. In the stages of precontemplation (Stage 1) and contemplation (Stage 2), the aim is to help adolescents gain a better understanding of the definition of IGD and statistics on studies of IGD in Iran and globally. The prevalence of IGD and the similarity of IGD with substance use disorders will be comprehensively described to the adolescents. Moreover, an explanation about the positive and negative consequences of online games on people’s health will be described.

##### Stage 3: Preparation

In the sixth week, a list of problems and difficult situations related to gaming will be provided for the adolescents to consider to prevent relapse. The adolescents will also write out different approaches for controlling online gaming and give a score of 1 to 10 out for all approaches presented. They will then choose an approach with respect to implementation and achievability.

##### Stage 4: Action

In the sixth week, adolescents will be encouraged to consider obstacles that may disrupt their plans. The adolescents will be asked to consider approaches to prevent them from being tempted to game online. Motivational videos will be provided to enhance adolescents’ confidence and motivation to perform better.

Factors that lead to maintaining good behavior and helping adolescents to achieve their goals are addressed at the beginning of the seventh session. Regarding the choice of a healthy lifestyle as a measure to prevent addiction to online games, it will be suggested that alternative behaviors could be tried such as having a sports program instead of going to gaming clubs, connecting with friends and attending parks and cinemas in groups, stress management training, and relaxation techniques.

##### Stage 5: Maintenance

In the seventh week, a worksheet of enjoyable activities will be provided to adolescents to prevent them from being tempted to play online games. Motivational videos will also be presented to enhance efforts to maintain their new behaviors. To enhance decisional balance, adolescents will receive information on the consequences of online games (both positive and negative consequences) including physical, mental, and emotional impacts in shorter and longer terms. In order to reduce stress and improve adolescents’ self-efficacy, training in relaxation techniques including body relaxation, breathing relaxation, yoga (as an audio file), mindfulness training, and mental imagery will also be used in the HAPPYTEEN app.

##### Active control condition

Eight educational sessions on “sleep hygiene” will be performed for the participants in the active control group. Sleep hygiene intervention was chosen for the control group based on a recent study on the targeted sample [1]. This study found that insomnia is prevalent among Iranian adolescents, and it can affect adolescents’ quality of life. Moreover, it was found that IGD severity was significantly associated with insomnia. Therefore, this intervention will be conducted to help adolescents in the active control group increase their quality of life and mental health.

The sleep hygiene condition includes video, text, and audio files that explain each section. In each training session, the purpose of that session and the previous content will be reviewed, and by providing complete explanations, the following content will be taught: sleep hygiene, study of sleep habits, sleep management, identifying incorrect patterns of behavior related to sleep and the impacts of environment, time, physical activity, and nutrition on sleep. The details of the intervention for the active control group are shown in Table 3.

**Table 3:** Intervention content for the active control group

Sessions	Content
First session	Introducing the program and aims of the training sessions, familiarity with the sleep process, scientific definition of sleep and sleep hygiene, studying the mechanisms of sleep and wakefulness, the effects of sleep on body organs and quality of life
Second session	A review of the contents of the previous session, the effect of sleep on the body, familiarity with the body's biological clock, the relationship between sleep cycle and wakefulness with age and normal sleep time during the day
Third session	Review of the contents of the previous session, review of sleep stages and NREM and REM cycles and review of changes in this cycle on body organs
Fourth session	A review of the contents of the previous session, review of sleep disorders, statistics on the prevalence of sleep disorders, definition of insomnia and characteristics of people with these disorders
Fifth session	Review of the contents of the previous session, review of sleep-disturbing factors such as identifying and modifying environmental factors affecting sleep patterns (e.g. sound, bed quality, temperature, etc.)
Sixth session	A review of the previous session, review of other factors that disrupt sleep, the relationship between nutrition and modification of eating habits with the benefit of adequate sleep, familiarity with the effect of exercise on experiencing good sleep
Seventh session	Review of the previous session, identifying and considering pre-sleep habits, the impact of technology on sleep, mentations and solutions to improve sleep
Eighth session	A summary and review of the sessions

### Sample size/power calculation

The sample size for the study was estimated based on a pilot study of 12 adolescents with IGD. The results of the pilot study showed that adolescents reported lower scores on the IGDS-SF9 at post-treatment follow-up (pre-treatment *M* = 34.59, *SD* = 16.91; post-treatment *M* = 25.37, *SD* = 17.94). However, 2 adolescents did not complete the post-treatment assessments. It is hypothesized that the app-based intervention will have at least a moderate mean effect size of 0.50 (Cohen’s *d*) compared to the control group. Using G*Power [61], sample size was calculated assuming a power of 90% for a two-group comparison analysis with three measures over time, the significance level of 0.05 and loss to follow-up of approximately 20%. The final sample size was set at 103 participants for each group.

### Assessments and outcomes

#### Data collection methods

**Fig. 2:**
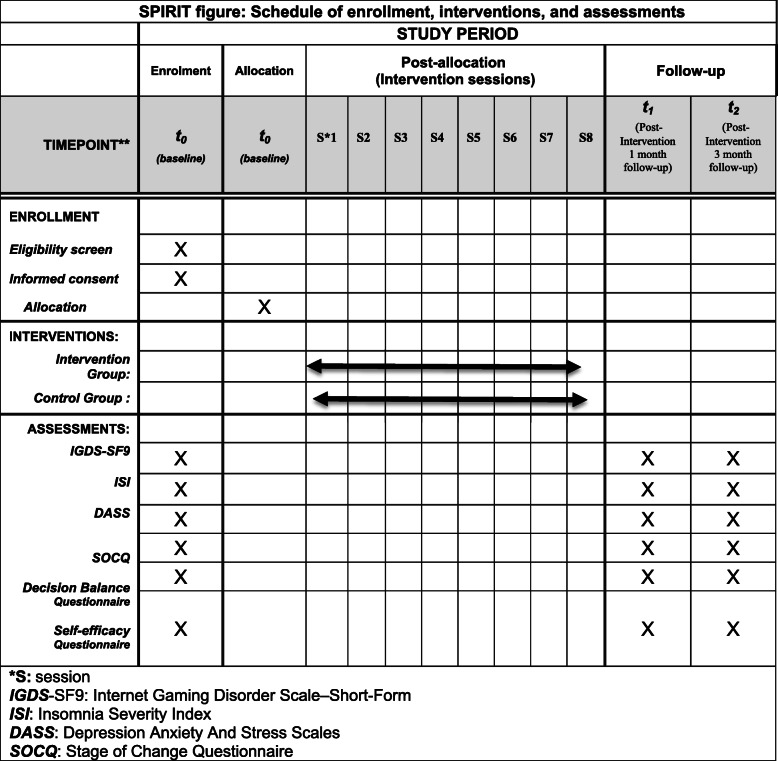
SPIRIT figure: schedule of enrollment, interventions, and assessments

Data collection from demographic information to measuring the severities of Internet Gaming Disorder (IGDS-SF9), insomnia (ISI), and depression, anxiety and stress (DASS-21), determining the status of the adolescents in stage of change in the TTM model (SOCQ), the degree of self-efficacy and decision-making balance in adolescents will be done with an online questionnaire. At the beginning of the study, participants will be assured that their information is and will remain confidential within the research team. Figure 2 provides a SPIRIT depiction of the schedule of enrollment, interventions, and assessments.

#### Sociodemographic variables

Demographic questions will include information on participants’ age, gender, educational level, type of school, parents’ education, type of gaming device (console, personal computer, cellphone, etc.), and duration of using online games in weekdays and weekends. This information will be collected by an online questionnaire.

##### Primary outcome measure

The primary outcome measure will be the IGDS-SF9. The IGDS-SF9 assesses the severity of IGD in adolescents [6, 62]. These activities include any game, whether online or offline performed through consoles, computers, laptops, tablets, and smartphones. The IGDS-SF9 has 9 items correspond to the nine IGD criteria defined by the DSM-5. All items are rated a five-point Likert type scale from 1 (never) to 5 (always). The total score is obtained by summing all 9 responses and range from 9 to 45 with higher scores indicating a higher risk of developing IGD. The Persian version of the *IGDS-SF9* was found to be highly valid and reliably among Iranian adolescents [62].

##### Secondary outcome measures

Secondary outcome measures will include the Depression, Anxiety and Stress Scale (DASS-21), Insomnia Severity Index (ISI), Stages of Change Questionnaire (SOCQ), Decisional Balance Scale, and Self-efficacy Scale.

##### The Depression, Anxiety, and Stress Scale (DASS-21)

To assess stress, anxiety, and depression in adolescents, the DASS-21 will be used [63]. The DASS-21 is a 21-item self-reported measure that consists of 3 subscales of stress, anxiety, and depression. Each subscale includes 7 items, and each item is rated on a four-point Likert type scale from 0 (did not apply to me at all) to 3 (applied to me very much, or most of the time). The subscale scores can be computed through the sum of the 7 responses and range from 0 to 21, with higher scores indicating greater anxiety, stress or depression. The validity and reliability of the Persian version of the DASS-21 was found to be acceptable in previous studies [64, 65].

##### Insomnia Severity Index (ISI)

The ISI is a brief self-reported instrument that measures adolescents' perceptions regarding the severity and the effects of insomnia. The ISI consists of seven items that are rated on a five-point Likert scale from 0 (no problem) to 4 (very severe problem). The total score is obtained by summing all 7 responses and the score ranges from 0 to 28 where 0–7 indicates absence of insomnia, 8–14 indicates subthreshold insomnia, 15–21 indicates moderate insomnia, and 22–28 indicates severe insomnia [66]. The Persian version of the ISI was found to be valid and reliable among both clinical and general populations of Iranians [67].

##### Stage of Change Questionnaire (SOCQ)

Stage of change will be measured using a single item: “Did you play online games for 20 hours or more a week during last month?”. Reponses options are as follows: “A. Yes, and I do not plan to reduce my digital game use. B. Yes, and I plan to reduce my digital game use in the next 6 months. C. Yes, and I plan to reduce my digital game use in the next 30 days. D. Not this month, but I have played digital games for less than 20 hours a week in the past 6 months. E. No, and I have not played digital games more than 19 hours a week in the past 6 months. F. No, and I have NEVER played digital games more than 19 hours a week”.

Based on the answers given, the participants will be classified into one of the five stages of change described in the present study: A = (Precontemplation), B = (Contemplation), C = (Preparation), D = (Action), E = (Maintenance), and F = (A participant that is unlikely to be struggling from IGD) [58].

##### Decisional balance

The Decision Balance Scale consists of 24 items that cover two subscales of the pros and cons of decreasing online gaming. The Decision Balance Scale has been developed based on previous studies of smoking and weight reduction [58]. Adolescents will be asked to rate their agreements for their decision to play or to avoid playing digital games. An example of Pro questions includes “Digital games are a good way for me to relieve stress.” An example of Con questions includes, “I am unable to focus on activities that are not related to digital games.” All items are rated on a five-point Likert scale from 1 (not important) to 5 (very important). The total subscale is obtained by summing all 12 responses with a range from 12 to 60 [58].

##### Self-efficacy Scale

The Self-efficacy Scale consists of 14 items that are rated on a five-point Likert scale from 1 = (strongly disagree) to 5 (strongly agree). This scale has two subscales assessing situational confidence and situational temptations. Each subscale has 7 items. The total subscale is obtained by summing all 7 responses and the total score ranges from 7 to 35 [58].

##### Assessment of adherence

Participants’ adherence to the interventions will be assessed by calculation the length of time using the app and number of log-ins using an online database. Moreover, the online database will be used to assess each participant’s viewing of each page and time spent on it.

### Intervention fidelity

All educational content were prepared under auditing of the first author (AHP) who is a professor in the field of health psychology and behavioral change. Also, all research processes including recruitment, intervention implementation, and assessments will be conducted under his supervision.

### Ancillary and post-trial care

After completing the follow-up assessments, if any individuals have IGD, they will be referred to a psychologist to receive further necessary counseling and or treatment.

### Statistical analysis

A blinded biostatistician will analyze the data using SPSS version 25 and MLwiN version 2.27 software. An intention-to-treat analysis will be used to compare the intervention effects between the active control and intervention groups. The Kolmogorov–Smirnov test will be used to assess normality distribution of study variables. Multilevel linear models (MLMs) using a random-effects model will be conducted to compare primary and secondary outcome measures across the two groups over baseline and at 1 month and 3 months after the intervention. Within-group intraclass correlations will be calculated, and a two-level mixed model will be used with adolescents nested in time.

## Discussion

The present study will aim to investigate the effectiveness of an educational intervention based on the TTM model and CBT in the treatment of IGD in adolescents. To the best of our knowledge, this study will be the first online intervention to aim to treat IGD and decrease the duration of online gaming and severity of IGD among adolescents. As described above, the present study uses a combination of TTM and CBT models for the intervention.

In general, cognitive, emotional, and physiological processes are involved in the development of addictions. These processes manifest in addictive behaviors. Therefore, theories and models of behavioral change have an important role in developing interventions [68, 69].

In the present study, in addition to using the CBT framework, the TTM is also used for developing the intervention. Using BCTs, the study attempts to increase motivation in adolescents in order to move from the early stages (i.e., precontemplation and contemplation) to later ones (e.g., maintenance). For people in the preparation and action stages, an attempt will be made to help them to the action and maintenance stages by using planning and increasing self-efficacy.

The app-based intervention was chosen for treating gaming disorder among adolescents for several reasons [1]. Despite help-seeking for a behavioral addiction typically being advantageous for affected people to obtain external assistance to facilitate dealing with the behavioral addiction, treatment-seeking is relatively infrequent in gaming disorder compared to other behavioral addictions [70]. Therefore, new modes of delivery (e.g., internet-based or app-based interventions) are warranted to increase dissemination. This approach is cost-effective and often easier to deliver compared to face-to-face delivery [2]. There is a concern for internet-related behavioral addictions with respect to internet-based modes of intervention (i.e., internet- or app-based interventions) may lead to worsening of the disorder in individuals. However, past studies have shown that such interventions can also be useful for internet-related behavioral addictions (including gaming addiction [71, 72]). Considering how people with internet-use concerns function with respect to internet use in general is important, particularly given the many aspects of daily life that involve internet use [3]. Due to the COVID-19 pandemic, many face-to-face interventions have shifted to online modes of delivery, given benefits that they offer.

The present study has some limitations. In the present study, only high-school students will be assessed. Therefore, the results of the study may not be generalized to younger age groups. Also, despite the use of a behavior-change model in the present study, the follow-up time (i.e., 3 months) to evaluate maintenance in students is relatively short. Nonetheless, the results of the present study should be useful to assess the efficacy of an online treatment for adolescents with IGD.

## Trial status

Current protocol version: 2.0 dated April 9, 2021. This trial has not yet started. It is anticipated to start 19 April 2021. Anticipated end date for recruitment: 31 May 2021.

### Dissemination policy

The authors will disseminate the study results via publishing one or more articles in peer-reviewed journals in related fields.

## Supplementary Information

**Additional file 1:** Screenshot of the HAPPYTEEN app.

## Abbreviations

BCTs: Behavior change techniques
CBT: Cognitive behavioral therapy
CONSORT: Consolidated Standards of Reporting Trials
DASS: Depression Anxiety Stress Scales
DSM-5: Diagnostic and Statistical Manual of Mental Disorders, version 5
GD: Gaming disorder
IGD: Internet gaming disorder
IGDS-SF9: Internet Gaming Disorder Scale–Short-Form
ISI: Insomnia Severity Index
RCT: Randomized controlled trial
SOC: Stage of change
