# Supplementary figures and images for: Effectiveness of a mobile app-based educational intervention to treat internet gaming disorder among Iranian adolescents: study protocol for a randomized controlled trial
Source: Trials. 2022 Mar 21;23:229. doi: 10.1186/s13063-022-06131-0 (PMC8935262; doi:10.1186/s13063-022-06131-0)

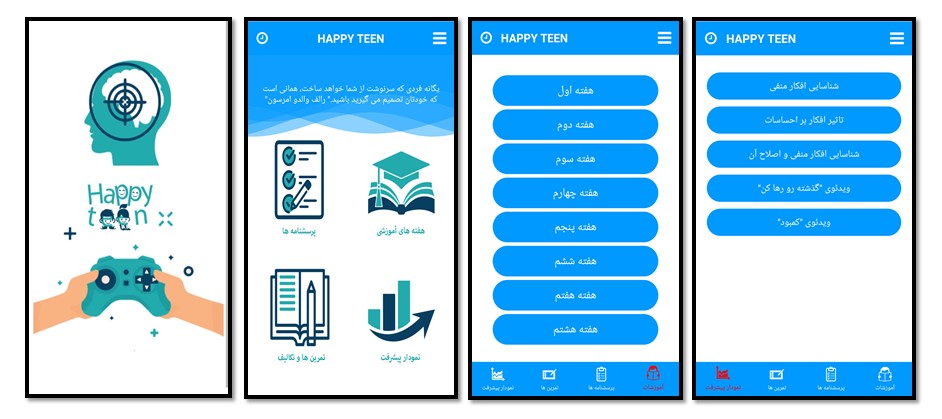


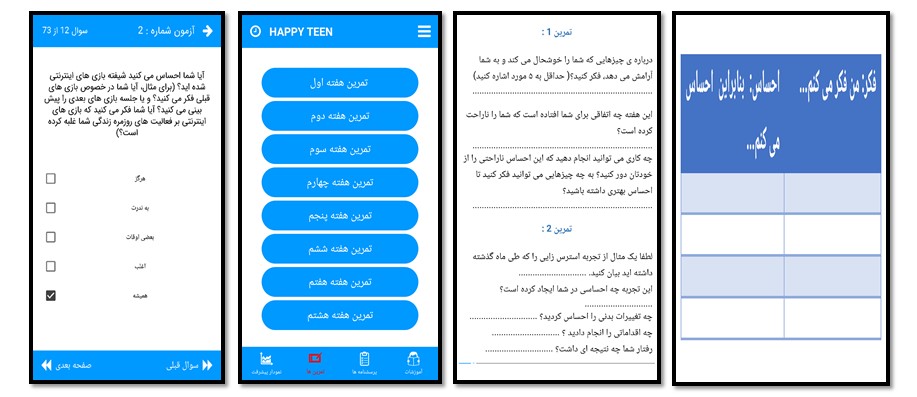


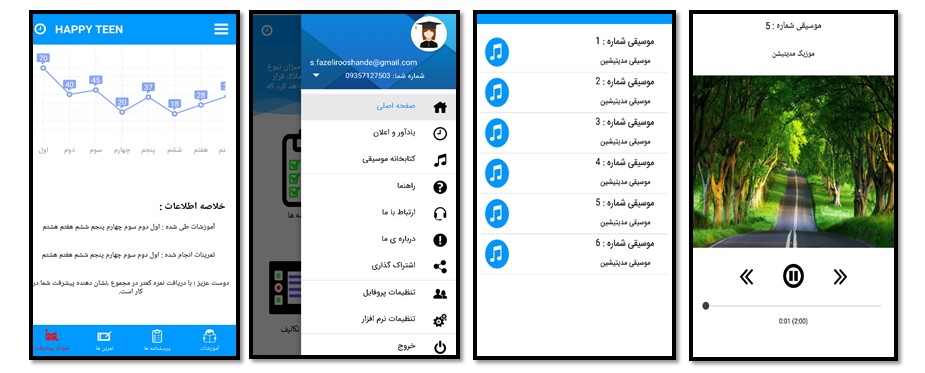

Supplement: Supplementary file 1 — Additional file 1: Screenshot of the HAPPYTEEN app. [file 13063_2022_6131_MOESM1_ESM.docx]
